# Detection of* Plasmodium* Aldolase Using a Smartphone and Microfluidic Enzyme Linked Immunosorbent Assay

**DOI:** 10.1155/2017/9062514

**Published:** 2017-09-06

**Authors:** Nikhil S. Gopal, Ruben Raychaudhuri

**Affiliations:** ^1^Lawrenceville School, Lawrenceville, NJ, USA; ^2^Medical College of Wisconsin, Milwaukee, WI, USA

## Abstract

**Background:**

Malaria control efforts are limited in rural areas. A low-cost system to monitor response without the use of electricity is needed.* Plasmodium* aldolase is a malaria biomarker measured using enzyme linked immunosorbent assay (ELISA) techniques. A three-part system using ELISA was developed consisting of a microfluidic chip, hand crank centrifuge, and a smartphone.

**Methods:**

A circular microfluidic chip was fabricated using clear acrylic and a CO_2_ laser. A series of passive valves released reagents at precise times based upon centrifugal force. Color change was measured via smartphone camera using an application programmed in Java. The microchip was compared to a standard 96-well sandwich ELISA.

**Results:**

Results from standard ELISA were compared to microchip at varying concentrations (1–10 ng/mL). Over 15 different microfluidic patterns were tested, and a final prototype of the chip was created. The prototype microchip was compared to standard sandwich ELISA (*n* = 20) using samples of recombinant aldolase. Color readings of standard ELISA and microfluidic microchip showed similar results.

**Conclusion:**

A low-cost microfluidic system could detect and follow therapeutic outcomes in rural areas and identify resistant strains.

## 1. Introduction

Effective strategies for the elimination of malaria are dependent upon tests that can provide early, accurate diagnosis. The gold standard has long been microscopic examination, but this is impractical in remote settings and requires specialized training. To circumvent this challenge, lateral flow based Rapid Diagnostic Tests (RDTs) were developed in the 1990s. Although these RDTs are cheap, fast, and easy-to-use, they do not provide a quantitative estimate of parasitic burden and are less sensitive for detection of* Plasmodium vivax* [1, 2]. More advanced diagnostic tests such as enzyme linked immunosorbent assays (ELISAs) do possess this capability but have similar drawbacks to microscopy, requiring expensive equipment, long incubation times, and trained staff. An easy-to-use, point of care, RDT like test with ELISA technology would likely have broad applicability in malaria diagnosis.

Microfluidics is a relatively new discipline that allows for fast, specific, and automated manipulation of small volumes of reagents. Several authors have described effective ELISAs using microfluidic techniques [3–7]. However, to our knowledge, no group has designed a microfluidics based ELISA for malaria. We were able to design an easy-to-use platform consisting of a circular chip to deliver proper reagents into a reaction chamber through channels and burst valves using a hand crank for energy and a companion smartphone camera and application to provide a simple read-out.

## 2. Objective

The primary objective was to create a system that would have similar sensitivity as a standard 96-well ELISA in detecting* Plasmodium* aldolase while using less reagent volume and cheaper equipment and without the need for AC power.

## 3. Materials and Methods

**Figure 1:**
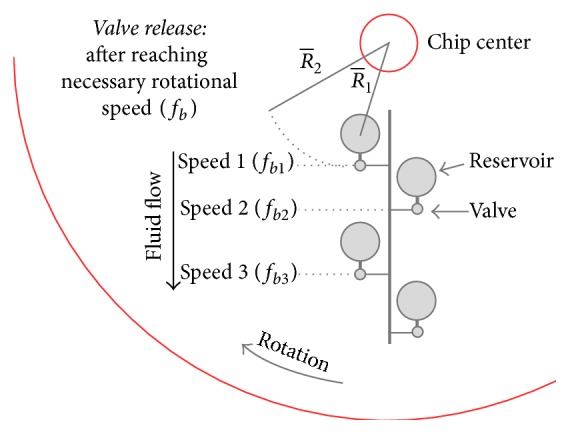
Controlled release of microfluidic reservoirs using passive valves. $f_{b}=\sqrt{\gamma \sin\theta /\pi^{2}2\rho \Delta R\overset{-}{R}d_{h}}$, where *f*_*b*_ is the burst force; Δ*R* the radius change between centers of both reservoirs; *ρ* is the liquid density; *γ* is the surface tension; *θ* is the angle of junction valve; $\overset{-}{R}$ = (*R*_1_ + *R*_2_)/2; *d*_*h*_ is the hydrodynamic diameter of channel connected to junction (4 *∗* cross section area of microchannel/contact line length).

We designed a circular microfluidic chip to deliver the antibodies and reagents in the correct order to a small reaction chamber. Microfluidic channels and burst valves directed flow without relying on mechanical valves or electricity, and the reagents were released based upon centrifugal force. Capillary burst valves are passive valves which require no moving parts. Fluid within a microchannel reaches a small junction between two reservoirs and is held in place by surface tension until a certain rotational speed is reached (Figure 1).

Approval from the local scientific safety review committee was received before experimentation began. Since no human tissue or patients were involved, no ethical committee approval was necessary.

### 3.1. Biomarker Selection

Dozens of potential biomarkers to measure presence of the* Plasmodium* species have been proposed over the past several decades. It is important to find a biomarker that is present in infected patients and does not rely on a human antibody response (which could take several months). One of the most promising of these potential biomarkers is* Plasmodium* aldolase [8]. Aldolase is an important enzyme in the pathway responsible for glycogen breakdown. Although aldolase is found in most animal species, the* Plasmodium* specific aldolase is antigenically dissimilar to humans and is found on the membrane of infected red blood cells.

### 3.2. ELISA

A sandwich ELISA was optimized using a capture antibody (Ab) and a conjugated secondary Ab. Mouse anti-*Plasmodium* aldolase monoclonal Ab was purchased from Santa Cruz Biotechnology. Recombinant* Plasmodium* aldolase from US Biological was used as antigen. The secondary Ab was a rabbit polyclonal anti-*Plasmodium* aldolase IgG and conjugated to horse radish peroxidase (Abcam). TMB substrate and phosphate buffered saline were purchased from Abcam. Tween-20 was used for assay buffers. Bovine serum albumin (5%) was used for all blocking (Amresco).

A 2-step checkerboard titration was used to determine the optimum dilutions of antibody and antigen. ELISA plates were read on a 96-well plate reader (Bio-Rad Model 550) at 450 nm after the stop solution was added. The optimal dilutions were as follows: capture Ab 1 : 8,000; antigen 1 : 64,000, and conjugated Ab 1 : 10,000. A hand crank centrifuge was purchased online and modified to accept a top plate with rubber stopper. The top plate was cut from acrylic using a CO_2_ laser. A battery powered tachometer was strapped to the side of the centrifuge to measure rotational speed.

### 3.3. Microfluidic Chip Fabrication

**Figure 2:**
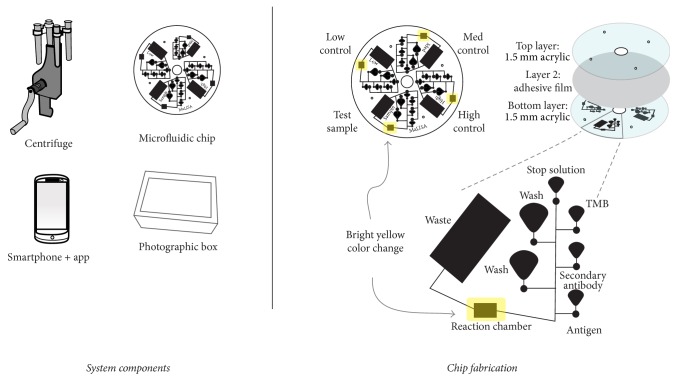
Microfluidic chip fabrication.

Optically clear acrylic sheets (1.5 mm) were purchased from McMaster-Carr. A 40-watt CO_2_ laser engraver (K40-Julong) was used. Designs were created in CorelDraw 12 and engraved. Chips were laminated using 3M-501FL adhesive. The dual layer chip was aligned using alignment holes. Flow was directed to a central channel and into a reaction chamber. After fluid left the reading chamber, it was sent to a waste reservoir (Figure 2).

### 3.4. Smartphone Camera Application

The application was programmed using Java and Android Studio IDE. A Nexus 6P smartphone running Android 6.0.1 was used. An app was programmed to calculate color intensity values when the user presses an on-screen button. The first three color intensity values are stored as control values (low, medium, and high), and the fourth is the unknown test sample. The app calculates a standard curve and extrapolates the value of the test sample. A copy of the source code can be found in https://github.com/ng4567/Malisav3.

### 3.5. Comparison between Standard ELISA and Microfluidic ELISA

**Figure 3:**
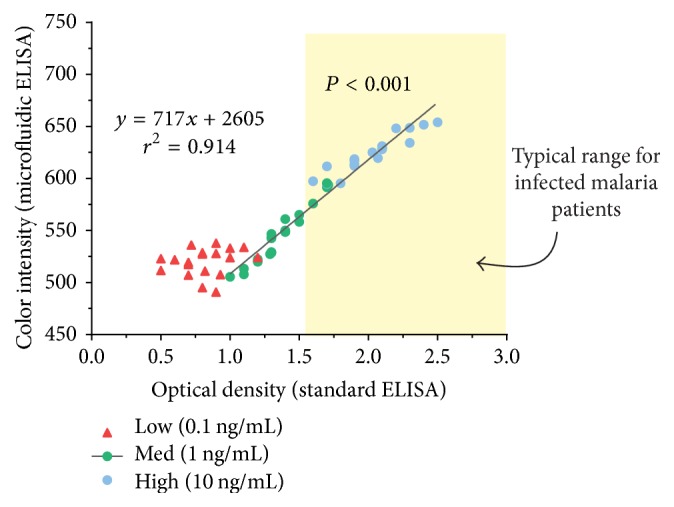
Microfluidic versus standard ELISA.

Three known concentrations of control aldolase solutions were prepared: low (0.1), medium (1.0), and high (10) ng/mL in PBS. At each concentration, twenty different assays were performed using standard ELISA and microfluidic methods. Optical density measurements were compared to the color readings from the smartphone. A regression line was fitted for each concentration (Figure 3).

### 3.6. Statistics

Statistical computations were performed using Graphpad Prism (version 6.02). A scatterplot was created and a Pearson product correlation was calculated. A two-tailed significance of 5% was used with the null hypothesis being that there was no relationship between optical density and app measured color intensity.

## 4. Results

The fitted regression line demonstrated a good relationship in the high and medium range (*r*^2^ = 0.914). However, the low concentrations present a problem with the color intensity readings from the smartphone. The optical density readings from the spectrophotometer were linear between optical densities of 1.0 to 3.0, but below 0.8 sensitivity of the smartphone app was limited.

Although the results within the low end of the range do not show linearity, it is expected that humans infected with malaria have aldolase concentrations approximately 2–4-fold higher (optical density greater than 1.6) [9].

## 5. Discussion

ELISA based tests offer important advantages over RDTs. Most importantly, they are quantitative assays, so they are capable of monitoring response to therapy, which is becoming increasingly important in the era of multidrug resistant malaria [10]. In addition, they tend to have greater sensitivities and specificities, especially for non-*falciparum* species of malaria [11]. Unfortunately, ELISA based detection has historically been somewhat impractical for widespread use. However, with the hardware described here, this need not be the case.

We present a method for providing quantitative results with point of care microfluidics technology. It is inexpensive, portable, and easy-to-use and does not require access to a local power grid. Given these characteristics, we believe our tool will be most useful for treatment follow-up in malaria endemic rural settings where access to electricity and qualified healthcare workers continues to be severely limited [12–14].

Although the results of our prototype are encouraging, much work needs to be done prior to large-scale implementation in the clinical setting. To increase the sensitivity of our assay, we are investigating alternative biomarkers to recombinant aldolase such as histidine rich protein 2 (HRP-2) and lactate dehydrogenase (LDH) [15]. Although aldolase is very well conserved amongst* Plasmodium* species, there are reports that HRP-2 based detection tests may have better sensitivity for malaria [16]. We are also in the process of evaluating the diagnostic utility of our assay using blood smear validated serum samples of uninfected, infected, and treated patients from malaria endemic regions. We will then determine optimal optical density cut-offs by receiver operating characteristics, at which point the assay will be available for field-testing.

The modular approach to the design of this ELISA allows for opportunities beyond the monitoring of treatment response. For instance, the flexibility of our platform allows for the rapid batch analysis of cohorts to detect rare infected patients, a strategy that will become more important as the world turns towards malaria elimination. This technology could also easily be applied to other antigens for detection of and/or treatment monitoring of other diseases.

In summary, we believe that the prototype we have developed has the potential to facilitate the diagnosis and treatment monitoring of patients in resource poor regions and represents a significant improvement over current ELISA based detection efforts.
